# A Combined Enrichment and Aptamer Pulldown Assay for *Francisella tularensis* Detection in Food and Environmental Matrices

**DOI:** 10.1371/journal.pone.0114622

**Published:** 2014-12-23

**Authors:** Elise A. Lamont, Ping Wang, Shinichiro Enomoto, Klaudyna Borewicz, Ahmed Abdallah, Richard E. Isaacson, Srinand Sreevatsan

**Affiliations:** 1 Department of Veterinary Population Medicine, University of Minnesota, St. Paul, Minnesota, United States of America; 2 Department of Veterinary Biomedical Sciences, University of Minnesota, St. Paul, Minnesota, United States of America; 3 Department of Biology, University of Utah, Salt Lake City, Utah, United States of America; 4 Molecular Ecology Group, Wageningen University, Dreijenplen 10, 6703HB, Wageningen, Netherlands; University of Houston, United States of America

## Abstract

*Francisella tularensis*, a Gram-negative bacterium and causative agent of tularemia, is categorized as a Class A select agent by the Centers for Disease Control and Prevention due to its ease of dissemination and ability to cause disease. Oropharyngeal and gastrointestinal tularemia may occur due to ingestion of contaminated food and water. Despite the concern to public health, little research is focused on *F. tularensis* detection in food and environmental matrices. Current diagnostics rely on host responses and amplification of *F. tularensis* genetic elements via Polymerase Chain Reaction; however, both tools are limited by development of an antibody response and limit of detection, respectively. During our investigation to develop an improved culture medium to aid *F. tularensis* diagnostics, we found enhanced *F. tularensis* growth using the spent culture filtrate. Addition of the spent culture filtrate allowed for increased detection of *F. tularensis* in mixed cultures of food and environmental matrices. Ultraperformance liquid chromatography (UPLC)/MS analysis identified several unique chemicals within the spent culture supernatant of which carnosine had a matching *m/z* ratio. Addition of 0.625 mg/mL of carnosine to conventional *F. tularensis* medium increased the growth of *F. tularensis* at low inoculums. In order to further enrich *F. tularensis* cells, we developed a DNA aptamer cocktail to physically separate *F. tularensis* from other bacteria present in food and environmental matrices. The combined enrichment steps resulted in a detection range of 1–10^6 ^CFU/mL (starting inoculums) in both soil and lettuce backgrounds. We propose that the two-step enrichment process may be utilized for easy field diagnostics and subtyping of suspected *F. tularensis* contamination as well as a tool to aid in basic research of *F. tularensis* ecology.

## Introduction

Increased global processing and distribution of food has raised awareness of food safety in regards to accidental or purposeful introduction of a biological contaminates into the food network [1], [2]. *Francisella tularensis* subsp. *tularensis*, a Gram-negative bacterium responsible for tularemia in a wide range of hosts, is categorized as a class A agent by the Centers for Disease Control and Prevention (CDC) due to its high infectivity, ease of dissemination and ability to cause disease [3], [4]. Current models of *F. tularensis* infectivity and dissemination concern aerosolization leading to pneumonic tularemia; however, tularemia may exist as oropharyngeal and gastrointestinal clinical forms due to oral exposure and/or ingestion of contaminated food or water [4]–[7]. Clinical presentation of oropharyngeal and gastrointestinal tularemia may include lesions in the oropharynx, draining lymph nodes, and gastrointestinal tract [5], [8]. Progression from oropharyngeal to pneumonic tularemia (aspiration) may occur due to bacteremic spread into the lungs [9], [10].

Traditional diagnostic tools for *F. tularensis* have been developed for patient samples and consequently rely on host responses, including serum antibodies [11]–[15]. Serodiagnostics for *F. tularensis* require antibody levels that are achieved after 10 or more days of disease and would provide minimal information about the source of infection and how to best manage a potential outbreak [5]. Availability of *Francisella tularensis* genomes and comparative analyses against other members of the *Francisella* genus have allowed researchers to use specific *F. tularensis* genes in diagnostic formats such as Polymerase Chain Reaction (PCR) and real-time PCR [16]–[22]. It is important to note that the gold-standard to validate *F. tularensis* detection using serology and various PCR platforms remains cultivation of the organism, which requires growth on cysteine or thioglycolate enriched medium and incubation times of 2–4 days at 37°C [5], [23]. Studies utilizing these tools have been widely applied to *F. tularensis* detection in patients and animal carcasses; however, few techniques have been reported for identification of *F. tularensis* in food and environmental matrices [24]–[27]. Inasmuch as the potential for biocontamination with *F. tularensis* and the presence of resident microbes, which may outcompete *F. tularensis* growth and act as PCR inhibitors, there remains a critical need for improved cultivation and unambiguous detection of *F. tularensis* in food and environmental matrices.

In this study, we report on the development of a two-step enrichment process for improved cultivation and detection of *F. tularensis* in lettuce and soil. This process first utilizes logarithmic-phase *F. tularensis* spent culture filtrate to supplement standard culture medium to enhance *F. tularensis* growth in the presence of resident bacteria from food and environmental matrices. Next, *F. tularensis* is further concentrated by physical separation from resident bacteria using a DNA aptamer cocktail capture assay.

Initial characterization of unique chemical entities found within the spent culture filtrate was carried out using UPLC/MS analysis with automated and manual database searches. Manual database searches identified carnosine as a chemical target that had similar *m*/*z* and retention time characteristics to those found by UPLC/MS. Addition of 0.625 mg/mL to conventional growth medium resulted in increased growth of *F. tularensis.* We propose that the application of the two-step enrichment process may be utilized in field diagnostics and molecular subtyping as well as extended to basic research in understanding the ecology of tularemia.

## Results

### *F. tularensis* spent medium increases the growth and detection of *F. tularensis* in mixed cultures of bacteria from food matrices

**Figure 1:**
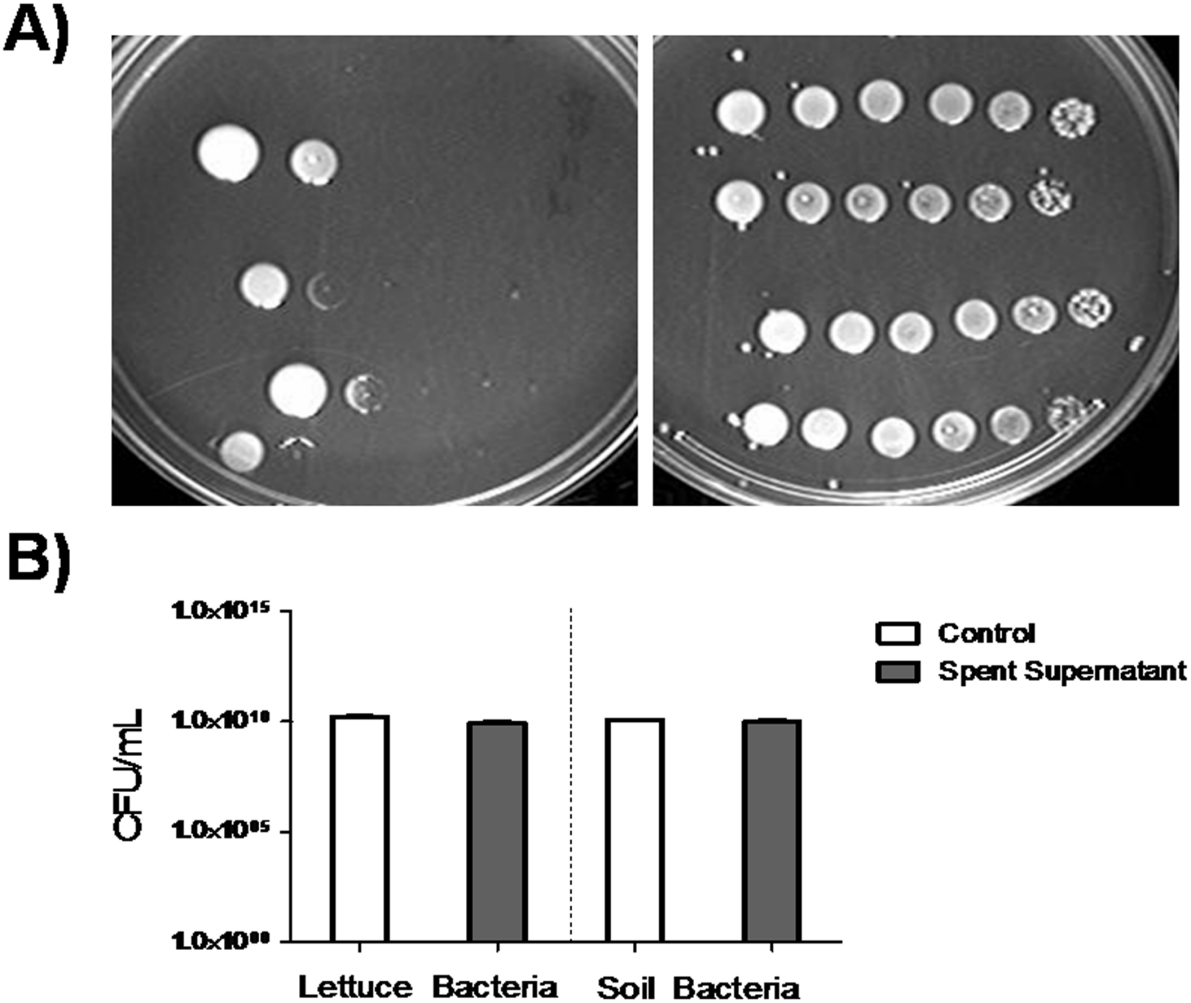
*F. tularensis* spent medium enhances growth and is specific for *F. tularensis*. **A)** *F. tularensis* was cultured for 24 h and diluted on TSA containing 0.1% L-cysteine (*left*) and TSA containing 0.1% L-cysteine and 10% spent medium (*right*). A ten-fold dilution series of *F. tularensis* was created (10^−1^–10^−6^, left to right). **B)** Lettuce bacteria were obtained from stomacher processing of 10 g of lettuce in 50 mL of TSB containing 0.1% L-cysteine. Soil bacteria were obtained from culturing of 1 g of soil, hay and dust in TSB containing 0.1% L-cysteine overnight at 37°C with shaking at 120 rpm. Bacteria from lettuce and soil sources were diluted to 10^−6^ and plated on TSA supplemented with 0.1% L-cysteine (control) and control agar supplemented with 10% *F. tularensis* spent filtrate. Samples were incubated overnight at 37°C and the CFU/mL was calculated. Legend: Control = white bar, Spent filtrate = medium grey bar.

Intracellular pathogens frequently grow slower, have a long lag time, or fail to grow in vitro compared to replication within the host cell [28]–[31]. This poses a significant challenge for disease and bioweapon diagnostics, which rely on specific detection ranges of pathogen cells, toxin and/or other protein concentrations. Detectable levels from low starting inoculums of *F. tularensis* are difficult to achieve in vitro. Various studies have shown that the spent culture medium of pathogenic bacteria, such as *Mycobacterium tuberculosis*, stimulated enhanced growth of dormant and small inoculum cultures [32]–[34]. More importantly, studies conducted by Halmann and colleagues have described the presence of a growth-initiation substance (GIS) in the culture filtrate using small inocula of virulent strains of *F. tularensis* [35], [36]. Therefore, we tested if the spent culture filtrate of *F. tularensis* vaccine strain would stimulate and enhance the growth of a small number of *F. tularensis* cell. Overnight cultures of *F. tularensis* were serially diluted and spotted on standard culture medium (TSA and 0.1% L-cysteine) supplemented with and without spent culture filtrate (Fig. 1A). *F. tularensis* cultured on spent culture filtrate resulted in robust growth at all dilutions (10^−1^–10^−6^) in contrast to standard culture medium, which only supported the growth of 10^−1^–10^−2^ dilutions (Fig. 1A).

**Figure 2:**
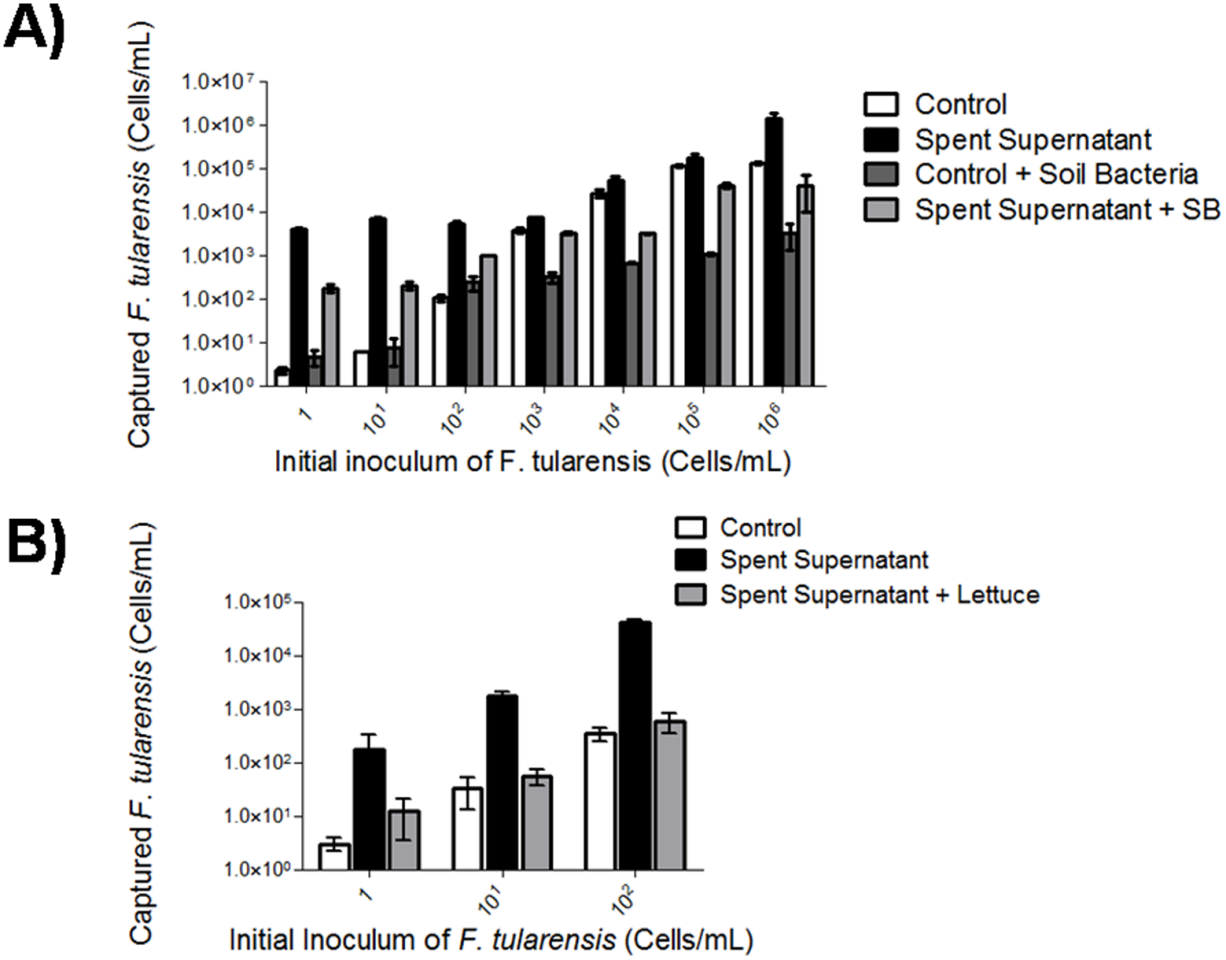
Spent filtrate supplementation results in increased detection of *F. tularensis* in mixed cultures. **A)** *F. tularensis* was spiked in a ten-fold serial dilution in 10^9 ^CFU/mL of soil bacteria or inoculated as a pure culture and incubated in control broth (TSB with 0.1% L-cysteine) or spent filtrate (composition: TSB and 0.1% L-cysteine and 10% spent culture filtrate) overnight at 37°C with 120 rpm shaking speed. **B)** *F. tularensis* (1–10^2^ cells/mL) was spiked in 10^9 ^CFU/mL of lettuce bacteria. Supplementation of TSB and 0.1% L-cysteine with spent culture filtrate resulted in *F. tularensis* detection at all inoculums (1–10^2^ cells/mL). Control medium combined with lettuce showed Cp values greater than 34 and were considered negative for *F. tularensis*. All samples were conducted in triplicate with technical triplicates. Legend: Control = white bar, Spent filtrate = black bar, Control and soil bacteria = dark grey bar, and Spent filtrate and soil/lettuce bacteria = light grey bar.

The main challenge in diagnostics and pre-analytical processing of *F. tularensis* in food and environmental matrices is the presence of other bacteria, which may interfere with *F. tularensis* growth [7]. Furthermore, the possibility remained that *F. tularensis* spent culture filtrate may stimulate inter-species growth of bacteria found in food and would consequently limit its’ utility as a diagnostic aid. To determine if *F. tularensis* spent culture medium promoted growth of other bacterial species, we compared the CFU/mL of bacteria found within lettuce and soil on spent filtrate supplemented medium and standard medium alone (Fig. 1B). We observed no difference in CFU/mL between standard medium supplemented with or without spent filtrate (Fig. 1B). However, the possibility remains that the spent culture filtrate may inhibit the growth of certain bacterial species, while other bacterial communities may continue to exist and fill the vacant niche. In fact, we did observe changes to colony phenotype when the spent culture filtrate was added to lettuce homogenates (*data not shown*). Next, we investigated our ability to detect *F. tularensis* by real-time PCR. Specifically, does the addition of spent culture filtrate in relation to the limit of detection and does the inclusion of soil and lettuce bacteria interfere with (Fig. 2)? *F. tularensis* cultured with spent filtrate showed a 2.5–3.5 fold increase in cells per mL at a starting inoculum of 1 bacterium per mL compared to standard medium alone (Fig. 2). Addition of soil bacteria to *F. tularensis* cultured with spent filtrate reduced the overnight growth of *F. tularensis* an average of 1.5 fold; however, a 2 fold increase was observed when compared against *F. tularensis* mixed with soil bacteria in standard medium (Fig. 2A). Given that the spent culture filtrate resulted in enhanced detection at low inoculums (1–10^2^ cells/mL), we focused on detection of *F. tularensis* at 1–10^2^ cells/mL spiked in lettuce bacteria (Fig. 2B). Supplementation of TSB and 0.1% L-cysteine with spent culture filtrate resulted in increased growth of *F. tularensis* mixed with lettuce bacteria at all inoculums (1–10^2^ cells/mL) in comparison to standard medium (Fig. 2B). It is interesting to note that unlike soil bacteria, inclusion of lettuce bacteria inhibited *F. tularensis* in control samples (Fig. 2B). Together these data confirm the findings presented by Halmann et al. and suggest that the increased growth of *F. tularensis* due to the addition of spent culture filtrate is unhindered by the presence of other bacteria [35].

### DNA aptamer cocktail captures *F. tularensis* in mixed cultures of bacteria

In order to further lower the range of detection, we generated DNA aptamers against *F. tularensis,* which may be applied to diagnostics in food matrices to achieve physical separation of *F. tularensis* from background bacteria. Aptamers, short RNA and single stranded DNA sequences, have high affinities to their selected receptors, show limited cross-reactivity to homologous targets, and may serve as pre-analytical tools and biosensors for food surveillance [37]–[41]. DNA aptamers against *F. tularensis* were enriched through 11 iterations of SELEX and 4 iterations of counter-SELEX. Confirmation of *F. tularensis* binding to the selected aptamer pool (from SELEX round 8) was determined by Southwestern blot analysis. The selected aptamer pool showed binding to 10^3^ cells of *F. tularensis* as opposed to the unselected aptamer library which could not detect the presence of *F. tularensis* at this concentration (*data not shown*). Sequencing of 188 transformants containing aptamer inserts revealed 10 redundant DNA motif sequence groups (S1 Fig.). A single aptamer (those with the highest number of repeat clones) were selected from each motif group (Table 1) for further testing against *F. tularensis*.

**Table 1:** Repeat DNA sequences and number of clones from sequenced aptamer pool^a^.

DNA Aptamer	Sequence (5′---3′)	No. of Repeats
FT17	CGCGTCAGAGGTGTGTCGGGGCTGTGTAGATCTACATGGG	17
FT12	CAGTCGCTTTCCGTTCTCCGGCAGGTTCATTGTGGTTTCG	12
FT11	CATATCAGGTCGTCACCGTAACAGAGCTCTCGCAATCACG	11
FT9	CAAAGGCAGCAGTAGCATGGCGATTTACATCAATTATTGG	9
FT8	CAATATCAGAAGTAGCGCGAAGGACGACATGTCAGGAAGG	8
FT7	CACACATCCTCGCAGCCTCGTACCTGATTCCAGTCTATTG	7
FT6	CCAGGAAGGCGAGAGCCGAGAAGCGATCCTTGGGTATAGG	6
FT5	CGGCATCCGTTCACGTACCTGTCCTAGTTATCACCGTTTG	5
FT5.1	CAAGCAGAGTTCCGAGACACAGTACCACACGCATATCCGG	5
FT4	CACACAGAGACGGTGAAGGCGCCGACCAGTTCCTAAAGAG	4

a Sequence aptamer pool belonged to the 11^th^ round of SELEX.

**Figure 3:**
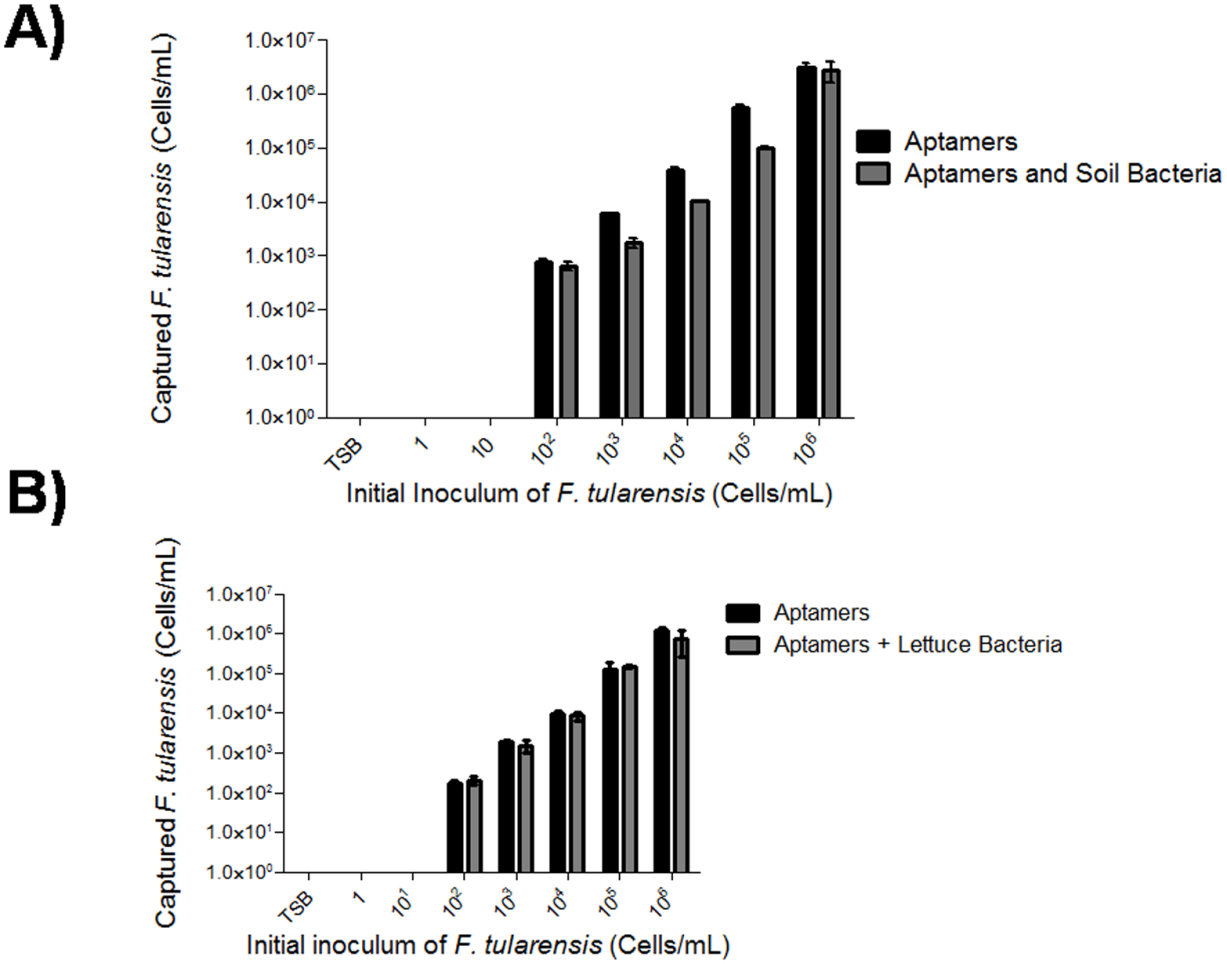
DNA aptamers against *F. tularensis* specifically capture *F. tularensis* in mixed bacteria samples. A biotinylated aptamer cocktail composed of 10 aptamers (0.4 pmol each) was bound to M-280 streptavidin Dynabeads. *F. tularensis* (1–10^6^ cells/mL) was separately mixed with **A)** 10^9 ^CFU/mL of soil bacteria or **B)** lettuce bacteria and aptamer cocktail beads or control beads (beads without aptamers) and incubated for 1 h at room temperature. Samples were denatured and the supernatant was subjected to real-time PCR analysis. Bead controls showed Cp values greater than 34 and were considered negative for *F. tularensis*. Legend: Aptamers = black bar, Aptamers and Soil Bacteria = dark grey bar.

Individual aptamers were tested for *F. tularensis* binding using *fopA* real-time PCR analysis. Cp values did not significantly differ from one another; therefore, the 10 aptamers were combined into a cocktail to increase binding efficiency (*data not shown*). The aptamer cocktail bound to M-280 Dynabeads was separately incubated with *F. tularensis* control and spiked lettuce or soil solutions. Using pure cultures of *F. tularensis* as well as mixed cultures (both soil and lettuce solutions), the range of *F. tularensis* cell capture was 10^2^–10^6^ cells/mL (Fig. 3A and B). Furthermore, *F. tularensis* spiked with lettuce bacteria was fully recovered at 10^2^–10^6^ cells/mL (Fig. 3B) while bead controls did not capture *F. tularensis* in pure or mixed cultures (Fig. 3). However, aptamers failed to capture *F. tularensis* at low inoculums (1–10 cells/mL) (Fig. 3). In summary, we have developed a DNA aptamer cocktail to enrich for *F. tularensis* from resident bacteria in food and environmental matrices at 10^2^–10^6^ cells/mL.

### Two-step enrichment process provides optimal recovery of *F. tularensis* in mixed cultures

**Figure 4:**
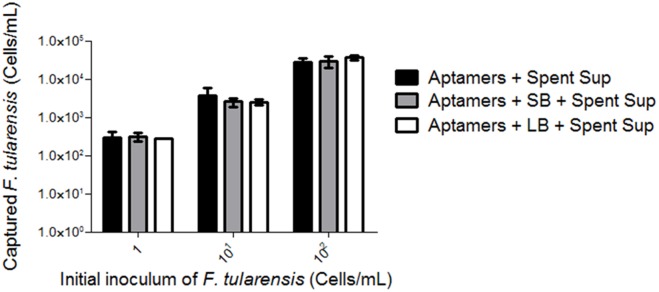
Combined enrichment with spent culture supernatant and DNA aptamer capture leads to complete isolation of *F. tularensis*. *F. tularensis* (1–10^2^ cells/mL) mixed samples in soil and/or lettuce homogenates were enriched for by overnight incubation with 10% spent culture supernatant and *F. tularensis* cells were subsequently captured by the DNA aptamer pool. Samples were denatured and the supernatant was subjected to real-time PCR analysis. Aptamers combined with either soil or lettuce bacteria alone (controls) showed Cp values greater than 34 and were considered negative for *F. tularensis*. Legend: Aptamers and Spent Sup (Spent supernatant) = black bar, Aptamers and SB and Spent Sup = grey bar, and Aptamers and LB and Spent Sup = white bar.

**I**n order to achieve *F. tularensis* recovery from small inoculums and physical separation in the presence of complex food and environmental matrices, we combined growth enhancement and DNA aptamer capture. Resident bacteria from lettuce and soil were utilized in this study as these sources represent food and environments that rabbits, reservoirs for *F. tularensis*, would encounter [8]. First, *F. tularensis* growth was increased by the addition of spent culture filtrate to standard medium during overnight incubation. This was subsequently followed with *F. tularensis* capture and separation from resident bacteria using the DNA aptamer cocktail. This two-step approach resulted in a lower limit of detection from a starting inoculum of 1 cell/mL (Fig. 4). Data suggests that enrichment with spent culture filtrate followed by physical separation and capture by the DNA aptamer cocktail can be utilized alongside current diagnostics as a pre-analytical processing tool.

### Carnosine yields enhanced growth for *F. tularensis* growth

**Figure 5:**
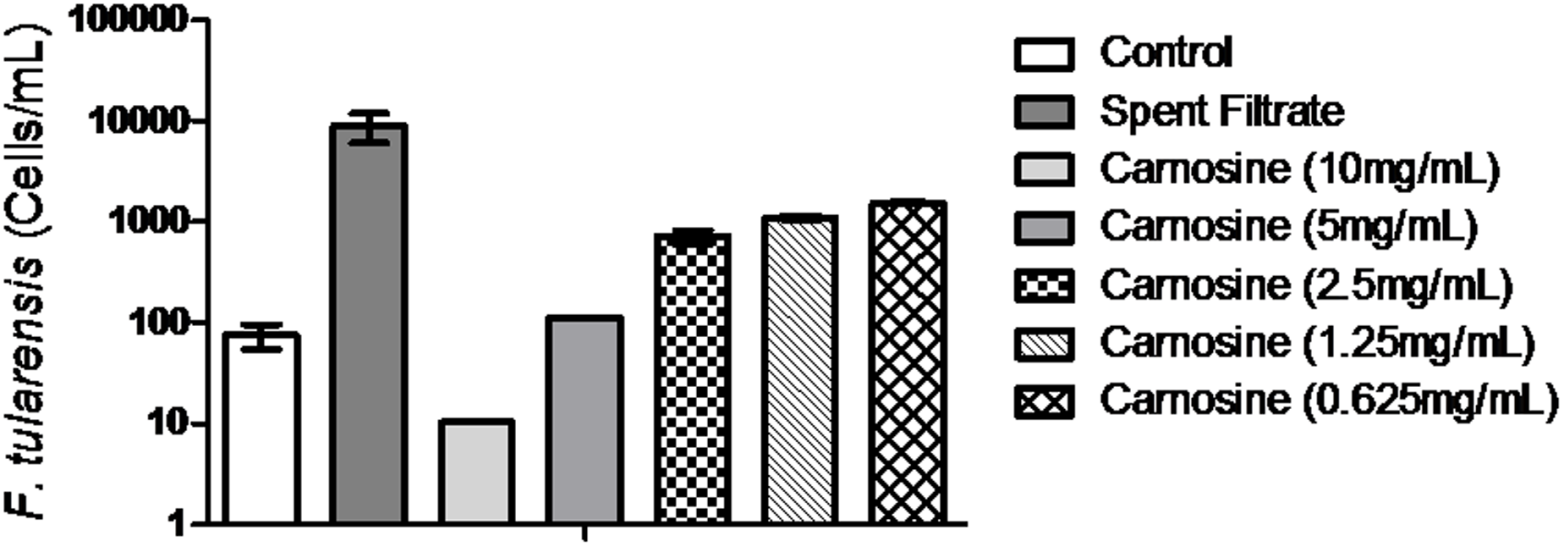
Carnosine increases *F. tularensis* growth. *F. tularensis* (10^2^ cells/mL) were incubated overnight at 37°C with agitation in control broth alone or supplemented with 10% spent culture filtrate or various concentrations of carnosine (0.625 mg/mL–10 mg/mL). DNA was extracted from all samples and analyzed by *fopA* real-time PCR. All samples were conducted in triplicate with technical triplicates. Legend: Control = white bar, Spent filtrate = dark grey bar, [carnosine = 10 mg/mL (light grey bar), 5.0 mg/mL (medium grey bar), 2.5 mg/mL (checkered pattern bar), 1.25 mg/mL (diagonal pattern bar) and 0.625 mg/mL (diamond pattern bar)].

Although the two-step enrichment process is an easy method that does not require specialized equipment for *F. tularensis* detection, it is not advisable to collect liter amounts of *F. tularensis* spent culture filtrate for multiple sample testing. Therefore, we sought to identify the GIS component within the *F. tularensis* spent supernatant to enable chemical synthesis and subsequent supplementation to control medium. We hypothesized that the GIS accumulates overtime; therefore, we selected spent culture filtrates at 0.5, 2, 8 and 24 h for UPLC/MS analyses. The analytical workflow to identify chemical targets specific growth enhancement entailed untargeted UPLC/MS profiling of spent culture filtrates; PCA analyses, chemometrics OPLS-DA; evaluation of extracted ion chromatograms (XICs) for each GIS specific ion; database searches to provide chemical identities of GIS features; mass accuracy confirmation and evaluation of fragmentation patterns for each target of MS simultaneously acquired high-collision energy mass spectra. PCA showed that the greatest separation between time points for both positive and negative ionization was achieved when comparing 0.5 and 2 h separately to 8 and 24 h post incubation (S2 Fig.). Therefore, the following comparisons were evaluated by OPLS-DA: 0.5 h v 8 or 24 h and 2 h v 8 or 24 h. OPLS-DA data were visualized by S-plots and those features unique to early (0.5 and 2 h) or late (8 or 24 h) were selected for further investigation. S-plot examples for positive and negative ionization and target selection for 2 v. 24 h are shown in S3 Fig. Similar S-plots were generated for 0.5 v 24 h and early time points v. 8 h (*data not shown*). Chemical entities with high confidence (high correlation) were defined as being unique to either early (−1.0) or late (1.0) time points and selected in lower left and upper right quadrants, respectively (S3 Fig.; selection represented by fuchsia boxes). Trend plots for each selected feature were created to determine abundance at comparison time points (S4A Fig.) with manual evaluation of XICs (S4B Fig.). Manual validation of tentative chemical entities (mined using Yeast, ChEBI, ChemSpider, KEGG, and LIPID MAPS databases) using UPLC/MS raw data and chemical standards failed. However, manual database searches (limited to parent ions and mass fractionation patterns of *m/z* of 397 and 297) as well as extensive literature based searches uncovered two potential pathways for enhanced growth 1) an autoinducer (such as N-acetyl-homoserine lactones (AHLs) or 2) carnosine, a chemical previously reported to cause enhanced growth and biofilm formation in *E. coli* [42]. Addition of commercially available AHLs to control broth did not yield enhanced growth of overnight cultures of *F. tularensis* (*data not shown)*. Carnosine supplementation at concentrations of 0.625–2.5 mg/mL to control broth resulted in increased growth of *F. tularensis* albeit to a lesser extent (10 fold) than the spent culture filtrate (Fig. 5). Higher concentrations of carnosine, particularly at 10 mg/mL, had a bacteriocidal effect on *F. tularensis* (Fig. 5).

## Discussion

Reliable and time-sensitive detection of a biological contaminant within the food network is essential to advise management and treatment procedures during a potential outbreak. The gold standard for microbe detection is successful cultivation of the agent in vitro; however, this remains a challenge for intracellular pathogens as many are either slow-growers or dormant in vitro. *F. tularensis*, a possible aerosol and biocontaminate in food, requires incubation for 2–4 days at 37°C when supplemented with cysteine, thioglycolate and/or blood. Furthermore, culture of *F. tularensis* in biological samples is also difficult to achieve in broth culture, unless a high starting inoculum is used.

Few studies have focused on the development of tools capable of detecting *F. tularensis* in food and the environment [24]–[27]. This is a significant concern given the potential of *F. tularensis* to contaminate food (accidently or deliberately). A 2009 study by Day and Whiting utilized a macrophage cell culture model (RAW 264.7) to isolate and enrich for *F. tularensis* in infant formula, liquid egg whites, and lettuce. Day’s and Whiting’s study was unique in the fact that they took advantage of *F. tularensis*’ niche cell, where the pathogen would efficiently replicate inside [24]. Once noninvasive resident bacteria were discarded from the cell culture medium and sufficient time was allowed for *F. tularensis* growth, real-time PCR analysis could identify as little as 10 CFU/mL of *F. tularensis* in infant formula and lettuce (5 h post-infection) and liquid egg whites (18 h post-infection) [24]. Although this method successfully enhanced the growth of and separated *F. tularensis* from food bacteria, it required specialized training in cell culture maintenance and access to equipment. This may not be feasible for field testing.

During the course of our investigation to create an improved cultivation medium for *F. tularensis*, we rediscovered initial experiments and subsequent observations reported by Halmann et al. [35], [36]. Halmann et al. report the presence of a Growth Initiating Substance (GIS) from low inocula of *F. tularensis* (strains Schu, Schu-M16, Vavenly, 425-F4G, 301I0, and LVS) that enhanced the growth of dormant *F. tularensis* cells when supplemented in traditional culture medium [36]. Similar to the Halmann studies, we have also shown that supplementation of standard medium with 10% spent culture filtrate results in *F. tularensis* enhanced growth in pure and mixed (lettuce and soil homogenates) cultures (Fig. 1A and Fig. 2). This data suggests that the majority of *F. tularensis* cells are composed of dormant cells capable of resuscitation with the addition of spent culture filtrate. Through a series of Sephadex gel filtration and ion-exchange chromatography, Halmann et al. characterized the GIS as having a low-molecular weight and heat stable at neutral pH but destroyable if heated in an acidic solution [36]. Furthermore, GIS was shown to form metal complexes (iron and copper complexes) and have enhanced activity when supplemented with ornithine [36]. Halmann and colleagues conjectured that ornithine may be a precursor to the GIS and that the GIS may be a sideramine or related to sideramine production. However, our analysis of the spent culture filtrate using UPLC/MS analysis and subsequent extensive database searches did not find any chemical entities related to sideramine function. UPLC/MS trend plots uncovered unique chemical signatures that had *m/z* ratios of 297 and 397; however, database searches were unable to provide a definitive identification. Manual database and literature searches related to bacterial growth and chemical entities with a *m/z* of 297–397 revealed AHLs or carnosine as suitable candidates. While available AHLs did not result in enhanced *F. tularensis* growth, carnosine (0.625 mg–2.5 mg/mL) promoted a 10-fold increase in *F. tularensis* cells/mL (Fig. 5). However, increased growth by carnosine did not fully recapitulate growth seen by the spent culture filtrate (Fig. 5). It is possible that other chemicals found within the spent filtrate act in concert with carnosine to increase *F. tularensis* growth or that the GIS component is a chemical related to carnosine and that additional side-chain components are responsible for the 10-fold difference. Higher concentrations of carnosine, specifically at 10 mg/mL, had a bacteriocidal effect (Fig. 5). This may be due to its ability to bind zinc and ferrous ions, which high carnosine concentrations may lead to iron toxicity or depletion of iron from the media [43]. Therefore, it is likely that *F. tularensis* may have a “Goldilocks” mechanism to regulate an appropriate amount of carnosine. These data suggest that carnosine functions both as a growth promoter and bactericide and that careful regulation by the bacterium must be conducted to ensure persistence. Further studies will need to revisit chemical entities identified by UPLC/MS to determine the exact GIS compound found in the spent supernatant and *F. tularensis* gene regulation in response to carnosine.

The second part of our *F. tularensis* enrichment process involves physical separation of *F. tularensis* from resident bacteria using DNA aptamer capture. Aptamers, short RNA or single-stranded DNA sequences, have high affinities to their target molecules and are sought after as an alternative to antibodies due to their ease of generation, amenability to work with various platforms and cost-effectiveness [38], [40], [44], [45]. We and others have shown that aptamers are effective tools to achieve separation of target bacteria from closely related microbes [37], [41], [46], [47]. We generated 10 redundant DNA aptamers, which were utilized in a cocktail to capture *F. tularensis* after growth enhancement. Physical separation of *F. tularensis* from resident bacteria by DNA aptamer capture increased the assay limit of detection and the recovered cell population was similar in number to that of pure cultures supplemented with spent culture filtrate (Figs. 3 and 4). Previous to our report, a study by Vivekananda and Kiel reported the generation of 25 unique DNA aptamer sequences specific for *F. tularensis* [48]. It is important to note that there are several key differences between the two study designs as well as data interpretation. In our study, we sequenced 188 transformants to reliably identify redundant motif sequences. Vivekananda and Kiel sequenced a total of thirty-five aptamer transformants and observed 10 duplications [48]. While we utilized the intact *F. tularensis* cell in rounds of SELEX and soil bacteria for counter-SELEX, Vivekananda and Kiel generated aptamers against a commercially available *F. tularensis* antigen and performed counter-SELEX against nitrocellulose membranes [48]. Although counter-SELEX would discard aptamers that bound to the nitrocellulose membrane, it is unclear if specificity for *F. tularensis* is greatly increased. However, the 2006 study does examine cross-reactivity of the 25 aptamers (combined in a cocktail) to lysozyme, albumin and *B. henselae* of which there is some reactivity to *B. henselae* [48]. Given that the optical density (405 nm) of the aptamers against the *F. tularensis* antigen in an Aptamer-Linked Immobilized Sorbent Assay (ALISA) was reported to be ∼0.1, it would be impossible to distinguish from *B. henselae* (∼0.3). DNA aptamers generated for this study failed to capture soil and lettuce bacteria and when combined with spent culture filtrate and real-time PCR had an increased detection range for *F. tularensis* (Fig. 4).

The combination of spent culture filtrate or carnosine and DNA aptamer capture as a two-part enrichment method for *F. tularensis* may be amendable for field diagnostics. For example, this method may be used in tandem with a portable real-time PCR device for rapid detection of *F. tularensis*. Furthermore, this method is advantageous to concentrate *F. tularensis* cells for subtyping, which may impact decision making in a potential outbreak. Benefits of such a diagnostic tool may be further extended in bench research investigating the ecology of *F. tularensis* by screening of animal carcass samples collected from multiple sites and varying years. Current investigations in our laboratory seek to determine if aptamer captured *F. tularensis* can be re-cultured. Pure cultures of *F. tularensis* isolates from the environment would allow investigators to pursue downstream studies that go beyond diagnostics.

## Materials and Methods

### Bacterial Culture

*F. tularensis* subsp. *holarctica* Live Vaccine Strain (LVS) NR-14 (Biodefense and Emerging Infections Research Resources Repositories, NAID, NIH; herein referred to as *F. tularensis*) was maintained in tryptic soy agar (TSA) supplemented with 0.1% L-cysteine at 37°C. *F. tularensis* was subcultured in tryptic soy broth (TSB) supplemented with 0.1% L-cysteine (Sigma-Aldrich, St. Louis, MO) at 37°C with 120 rpm shaking. Environmental bacteria (herein referred to as soil bacteria) were obtained from one gram (g) of soil, hay and dust and cultured in TSB and diluted to 10^−1^ in TSB containing 0.1% MgSO_4_^.^ 7H_2_O to represent low and high nutrition conditions at room temperature and 37°C under constant shaking at 120 rpm. Lettuce bacteria were cultured from bagged lettuce purchased from a local grocery store. Approximately 10 g of lettuce were mixed with 50 mL of TSB supplemented with 0.1% L-cysteine inside a sterile Stomacher 400 classic bag (Seward Laboratory Systems Inc., Port Saint Lucie, FL) and homogenized for 2 min at high speed in a Stomacher. Homogenate was subcultured at 10^−1^ in TSB supplemented with 0.1% L-cysteine at 37°C with 120 rpm shaking.

### *F. tularensis* Growth Enrichment

*F. tularensis* was grown as described overnight at 37°C with shaking at 120 rpm. Upon completion of incubation, *F. tularensis* was pelleted at 7000*×g* for 10 min and the supernatant was filtered through a 0.22 µM syringe-driven filter (EMD Millipore, Billerica, MA) to remove any residual bacteria. An overnight culture of *F. tularensis* was serially diluted ten-fold and spotted on TSA supplemented with 0.1% L-cysteine (control) or control medium with 10% (v/v) spent filtrate, carnosine (0.625 mg/mL–10 mg/mL) (Sigma-Aldrich, St. Louis, MO), and N-Acyle-homoserine lactones (AHLs) (Sigma-Aldrich, St. Louis, MO). All samples were incubated overnight at 37°C and differences in growth were recorded the following day. The number of *F. tularensis* cells per mL were determined by *fopA* real-time PCR analysis described below.

### Selection of DNA aptamers against *F. tularensis* using Systemic Evolution of Ligands by Exponential Enrichment (SELEX)

Aptamer candidates against *F. tularensis* were selected using a SELEX protocol. Briefly, a modified single-stranded aptamer library (WAP40m) consisting of a 40-mer randomized regions flanked by constant-primer binding regions (Integrated DNA Technologies, Inc., Coralville, IA) underwent four rounds of counter-SELEX iterations. The aptamer library was incubated for 30 min with 10^8 ^CFU/mL of soil bacteria (previously cultured overnight at both 25°C or 37°C) in TSB containing 0.1% L-cysteine at room temperature with gentle shaking (Labquake tube shaker) [45], [49]. Aptamers bound to soil bacteria were discarded and the filtrate was collected, which was used for positive rounds of SELEX against *F. tularensis*. Positive SELEX iterations involved exposure of the remaining aptamer library to 10^8^ cells/mL of *F. tularensis* at room temperature with gentle shaking for 30 min. Unbound or weak-binding aptamers were removed from further rounds of SELEX by washing cells 6 times in 1X PBS containing 0.025% Tween-80 and twice with nuclease-free water. Aptamers bound to *F. tularensis* cells were resuspended in 100 µL of nuclease-free water and subjected to Polymerase Chain Reaction (PCR) after every round of SELEX to amplify aptamer candidates. PCR was performed using a biotin-labeled on the primer’s anti-sense strand (WP20R1; 5 pmol), an unlabeled primer (WP20F1; 5 pmol), 2X Hotstar taq polymerase (Qiagen, Valencia, CA) and the following program: 95°C for 15 min, followed by 15 cycles of 95°C for 30 s, 63°C for 30 s, and 72°C for 7 min (Table 1). In order to enrich for aptamer sense strands needed for further rounds of SELEX, amplicons were applied as templates at 1∶10 and 1∶25 dilutions for asymmetric-touchdown PCR using 30 pmol of WP20F1 and 1.2 pmol of biotin labeled WP20R1. The asymmetric-touchdown program was 95°C for 15 min, followed by 9 cycles of 95°C for 15 s, 72°C for 15 s (gradually decreasing by 1°C each cycle), and 72°C for 15 s, followed by 11 cycles of 95°C for 15 s, 63°C for 15 s, and 72°C for 15 s, and a final extension at 72°C for 3 min. PCR amplicons were pooled and anti-sense strands were removed using a snap cool method with 50 µL of Dynabeads M-280 streptavidin coated beads (Invitrogen, Carlsbad, CA). The amplicon sense strand was applied to the remaining rounds of SELEX. A total of 11 SELEX iterations were performed.

### Cloning, sequencing and characterization of DNA aptamers against *F. tularensis*

Four hundred µL of PCR amplicons from the 11^th^ round of SELEX were purified using the MiniElute PCR purification kit (Qiagen, Valencia, CA). The purified PCR product was cloned into the TA-TOPO pCR 2.1 vector (Invitrogen, Carlsbad, CA) and later transformed into TOP10 chemically competent *E. coli* cells per manufacturer’s instructions. Transformed TOP10 *E. coli* cells (100 µL) were plated on Luria-Bertani agar containing ampicillin (50 µg/mL) and X-gal. Successful transformants were selected based on blue/white screening for disruption of *lacZ*. Colony PCR was performed on selected transformants using M13 primers and the following PCR conditions: 95°C for 15 min and 30 cycles of 95°C for 30 s, 56°C for 30 s, 72°C for 1 min, and final extension for 72°C for 5 min (Table 2). One hundred and eighty-eight transformants with the correct amplicon size were sequenced and analyzed for redundancy using Sequencher (GeneCodes, Ann Arbor, MI) and CLUSTAL W in MEGA 4.0 (http://www.megasoftware.net/mega4/mega.html) (Table 2 and S1 Fig.). Redundant aptamers were further characterized using the m-fold program (http://mfold.rna.albany.edu/?q=mfold/DNA-Folding-Form) available from the RNA Institute.

**Table 2:** Primers used in this study.

Primer	Sequence (5′---3′)
WP20F1	AGTGCAAGCAGTATTCGGTC
WP20R1	TAAAGCTGATGCGTGATGCC
M13F	GTTTTCCCAGTCACGAC
M13R	CAGGAAACAGCTATGAC
*fopA_*F	GTTCAAGGTGCTTGGATG
*fopA_*R	TGCAACAGCGCTAAGAGTTTT

### Spike and recovery of *F. tularensis*

An overnight broth culture of *F. tularensis* was washed three times in PBS, serially diluted ten-fold (1–10^6^ cells/mL) in TSB supplemented with 0.1% L-cysteine and spiked in 10^9 ^CFU/mL of soil or lettuce bacteria (homogenized solutions) or medium alone. Dilutions in medium alone and those mixed with soil or lettuce bacteria were supplemented with 10% filtered spent medium. Control samples did not include supplementation with spent filtrate. All samples were incubated overnight at 37°C with shaking at 120 rpm. After incubation, samples were pelleted at 8,000 rpm and filtrates were discarded. Genomic DNA was extracted from samples using the DNeasy blood and tissue kit (Qiagen, Valencia, CA) per manufacturer’s instructions. DNA was treated with 4.0 µL of RNase A (Qiagen, Valencia, CA) for 2 min at room temperature. Genomic DNA was suspended in 50 µL of nuclease-free water. All treatments were conducted in triplicate. All experiments were conducted in triplicate. Genomic DNA was stored at −20°C until real-time PCR analysis.

### Spike and aptamer cocktail recovery of *F. tularensis*

An overnight broth culture of *F. tularensis* was prepared and spiked in soil or lettuce bacteria (homogenized solutions) or medium alone as described above. Approximately 0.4 pmol of each selected biotin tagged aptamer (Table 1) was mixed together as a cocktail and bound to Dynabeads M-280 streptavidin coated beads (Invitrogen, Carlsbad, CA) using a modified protocol [38]. Briefly, 25 µL of M-280 streptavidin beads were loaded into individual wells in a 96 well PCR plate, washed 3 times in PBS and incubated with the biotin labeled aptamer cocktail overnight at 37°C with shaking at 200 rpm. After incubation, aptamers bound to M-280 streptavidin beads were washed thrice in 200 µL of PBS and blocked for 2 h at room temperature with shaking at 200 rpm using 100 µL of PBS containing 0.05% Tween-80 and 0.05% bovine serum albumin (BSA). Aptamer M-280 streptavidin beads were later washed twice in 200 µL of PBS and once with 200 µL of nuclease-free water. One hundred µL of *F. tularensis* control and spiked samples (soil or lettuce bacteria) were mixed separately in individual wells containing aptamer M-280 beads and incubated for 1 h at room temperature with 200 rpm shaking. Aptamer M-280 beads were washed 5 times in 200 µL of PBS containing 0.05% Tween-80 and thrice in 200 µL of nuclease-free water and resuspended in 100 µL of nuclease-free water. In order to release the genomic DNA from captured bacterial cells, they were heated to 95°C for 10 min. All treatments were conducted in triplicate and each experiment was performed thrice.

### Two-step enrichment of *F. tularensis* in Mixed Bacterial Cultures

An overnight culture of *F. tularensis* was washed thrice in PBS and diluted to 1–10^2^ cells/mL in TSB supplemented with 0.1% L-cysteine. *F. tularensis* dilutions were either mixed in medium with or without 10% spent culture filtrate alone or with 10^9 ^CFU/mL of lettuce or soil bacteria. All samples were incubated overnight at 37°C with shaking at 120 rpm and later mixed separately with aptamer M-280 streptavidin beads. All treatments were conducted in triplicate and each experiment was performed thrice.

### Real-time PCR Analysis of *F. tularensis* in Pure and Mixed Bacterial Cultures

Real-time PCR of the *fopA* gene was performed using *F. tularensis* spiked with soil or lettuce bacteria and control samples using a Lightcycler 480 Probes Master Mix and Universal ProbeLibrary Probe #10 (Roche, Indianapolis, IN) per manufacturer’s recommendations (Table 2). All samples were analyzed on the Roche Lightcycler 480II and corresponding software (Roche, Indianapolis, IN). The following real-time PCR conditions were applied: 95°C for 5 min and 45 cycles of 95°C for 10 s, 50°C for 30 s and 72°C for 1 s. Primers were designed using the Universal ProbeLibrary Assay Design Center (https://www.roche-applied-science.com/). The number of cells/mL was calculated for each sample based on a *F. tularensis* cells/mL standard curve. The standard curve was generated by a ten-fold serial dilution of *F. tularensis* genomic DNA with a known cells/mL (10–10^7^ cells/mL). Cross-point (Cp) values were calculated using the second derivative maximum method available in the 480 II lightcycler software (Roche, Indianapolis, IN) for each dilution in the standard curve and for all test samples [50]. Cp values from the standard curved ranged 13–34 cycles. Sample Cp values outside of the standard curve range were designated as negative for *F. tularensis*. T_m_ Calling analysis was used to verify negative samples [50]. In the case of a negative sample, T_m_ Calling results showed two-shouldered or no melting peaks. The former indicated the production of primer dimers. All samples were conducted in triplicate.

## Supporting Information

S1 Fig **DNA Aptamer Sequence Motifs.** The DNA aptamer pool was sequenced after the 11^th^ round of SELEX and sequences were aligned using CLUSTAL W in MEGA 4.0. Ten repeated motifs were identified and 1 DNA aptamer was selected from each motif group for further characterization.
(PDF)

S2 Fig **Principal Component Analyses (PCA) of** ***F. tularensis*** **spent culture filtrate.** MarkerLynx software was used to generate PCA plots of UPLC/MS data. PCA plots include positive (*right*) and (*negative*) ionization at 0.5 h (blue circles) v 24 h (seafoam green) and 2 h (blue circles) v 24 h.
(TIFF)

S3 Fig **OPLS-DA Generated S-Plots.** Chemometric S plot resulting from OPLS-DA of UPLC/MS analysis of *F. tularensis* spent culture filtrates at 2 h versus 24 h with positive and negative ionization. Similar S-plots were generated for 0.5 h versus 24 h. The y-axis represents correlation (confidence; time point specific), while the x-axis represents the coefficient (specificity). The selection boxes represent features that are highly specific to each time point. Selection boxes are noted in fuchsia.
(TIFF)

S4 Fig **UPLC/MS Characterization.** A) A chemometric trend plot depicting the relative abundance of the chemical entity found at 0.5 h and 24 h. B) Extracted ion chromatogram (XIC) for *m/z* 397.2205.
(TIFF)
