# Supplementary figures and images for: A Combined Enrichment and Aptamer Pulldown Assay for Francisella tularensis Detection in Food and Environmental Matrices
Source: PLoS One. 2014 Dec 23;9(12):e114622. doi: 10.1371/journal.pone.0114622 (PMC4275185; doi:10.1371/journal.pone.0114622)

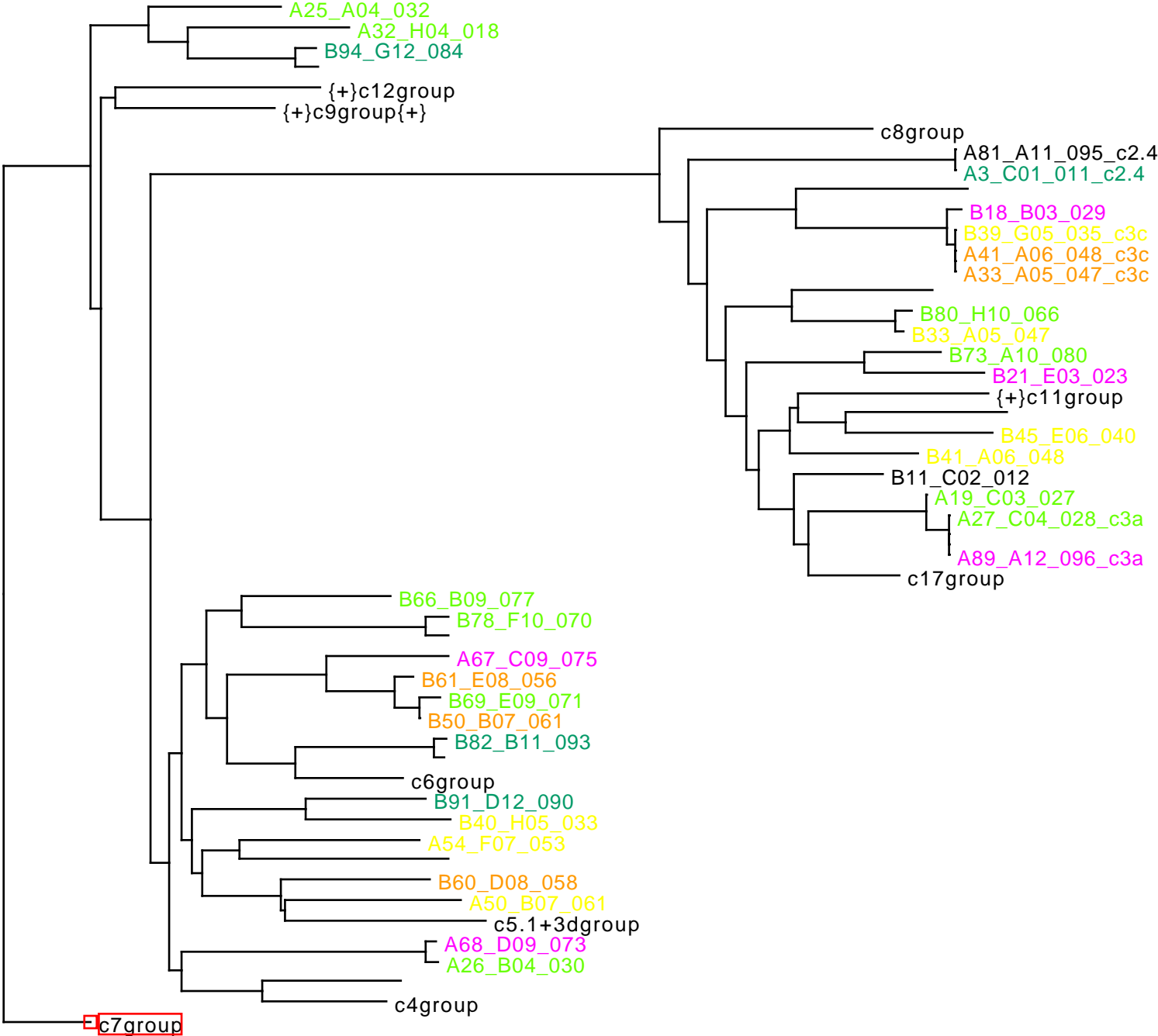

Supplement: S1 Fig — DNA Aptamer Sequence Motifs. The DNA aptamer pool was sequenced after the 11th round of SELEX and sequences were aligned using CLUSTAL W in MEGA 4.0. Ten repeated motifs were identified and 1 DNA aptamer was selected from each motif group for further characterization. (PDF) [file pone.0114622.s001.pdf]

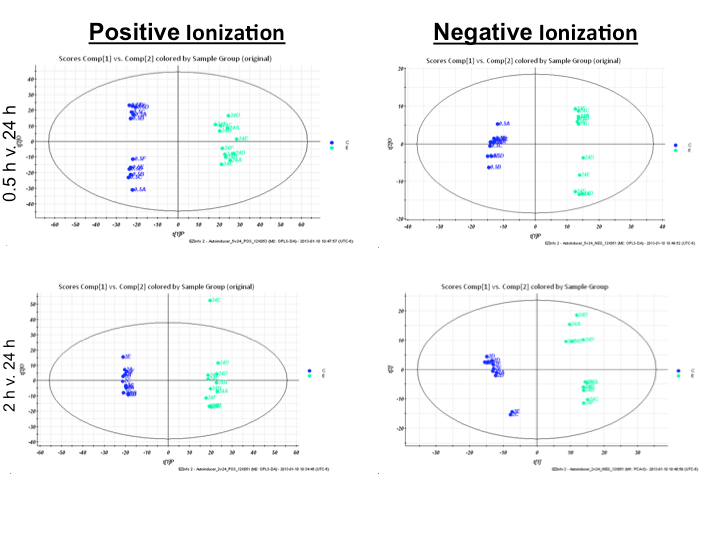

Supplement: S2 Fig — Principal Component Analyses (PCA) of F. tularensis spent culture filtrate. MarkerLynx software was used to generate PCA plots of UPLC/MS data. PCA plots include positive (right) and (negative) ionization at 0.5 h (blue circles) v 24 h (seafoam green) and 2 h (blue circles) v 24 h. (TIFF) [file pone.0114622.s002.tiff]

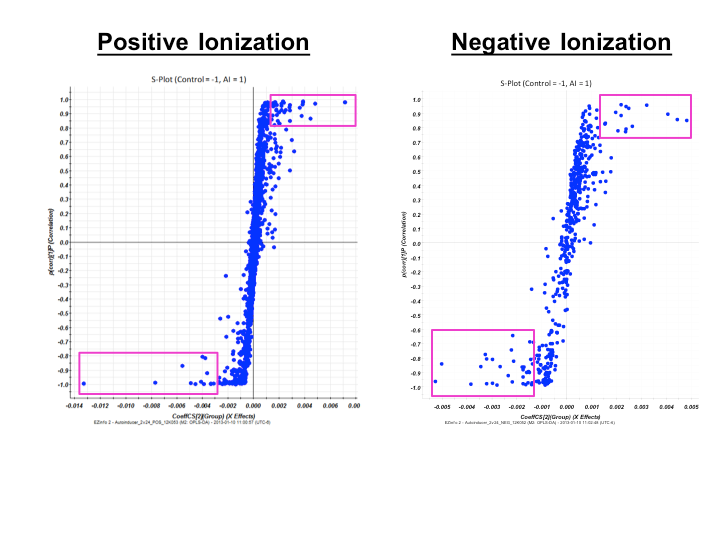

Supplement: S3 Fig — OPLS-DA Generated S-Plots. Chemometric S plot resulting from OPLS-DA of UPLC/MS analysis of F. tularensis spent culture filtrates at 2 h versus 24 h with positive and negative ionization. Similar S-plots were generated for 0.5 h versus 24 h. The y-axis represents correlation (confidence; time point specific), while the x-axis represents the coefficient (specificity). The selection boxes represent features that are highly specific to each time point. Selection boxes are noted in fuchsia. (TIFF) [file pone.0114622.s003.tiff]

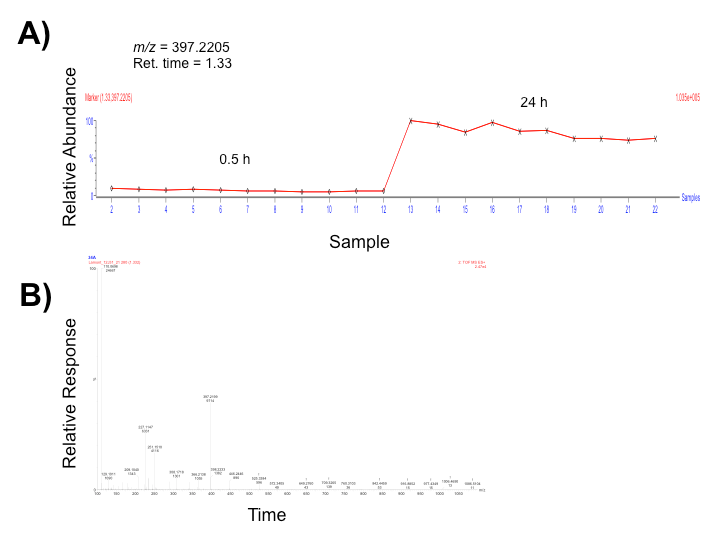

Supplement: S4 Fig — UPLC/MS Characterization. A) A chemometric trend plot depicting the relative abundance of the chemical entity found at 0.5 h and 24 h. B) Extracted ion chromatogram (XIC) for m/z 397.2205. (TIFF) [file pone.0114622.s004.tiff]
